# Increased Incidence of Tuberculosis in Zimbabwe, in Association with Food Insecurity, and Economic Collapse: An Ecological Analysis

**DOI:** 10.1371/journal.pone.0083387

**Published:** 2014-02-05

**Authors:** Stephen J. Burke, Elliot Lass, Paul Thistle, Lovemore Katumbe, Arif Jetha, Dan Schwarz, Shelly Bolotin, R. D. Barker, Andrew Simor, Michael Silverman

**Affiliations:** 1 University of Ottawa, Ottawa, Canada; 2 University of Toronto, Toronto, Canada; 3 Karanda Hospital, Mount Darwin, Zimbabwe; 4 The University of Zimbabwe, Harare, Zimbabwe; 5 The Howard Hospital, Glendale, Zimbabwe; 6 The Dalla Lana School of Public Health, Toronto, Canada; 7 Brigham and Women's Hospital and Children's Hospital of Boston, Boston, Massachusetts, United States of America; 8 Murambinda Mission Hospital, Murambinda, Zimbabwe; 9 Kings College Hospital, NHS Foundation Trust, London, United Kingdom; 10 Lakeridge Health Corp, Oshawa, Canada; Johns Hopkins Bloomberg School of Public Health, United States of America

## Abstract

**Background:**

Zimbabwe underwent a socioeconomic crisis and resultant increase in food insecurity in 2008–9. The impact of the crisis on Tuberculosis (TB) incidence is unknown.

**Methods:**

Prospective databases from two mission hospitals, which were geographically widely separated, and remained open during the crisis, were reviewed.

**Results:**

At the Howard Hospital (HH) in northern Zimbabwe, TB incidence increased 35% in 2008 from baseline rates in 2003–2007 (p<0.01) and remained at that level in 2009. Murambinda Hospital (MH) in Eastern Zimbabwe also demonstrated a 29% rise in TB incidence from 2007 to 2008 (p<0.01) and remained at that level in 2009. Data collected post-crisis at HH showed a decrease of 33% in TB incidence between 2009 to 2010 (p<0.001) and 2010/2011 TB incidence remained below that of the crisis years of 2008/2009 (p<0.01). Antenatal clinic HIV seroprevalence at HH decreased between 2001(23%) to 2011(11%) (p<0.001). Seasonality of TB incidence was analyzed at both MH and HH. There was a higher TB incidence in the dry season when food is least available (September-November) compared to post harvest (April-June) (p<0.001).

**Conclusion:**

This study suggests that an epidemic of TB mirrored socioeconomic collapse and recovery in Zimbabwe. The seasonal data suggests that food security may have been associated with TB incidence both annually and during the crisis in this high HIV prevalence country.

## Background

Zimbabwe underwent an economic collapse characterized by political crises, hyperinflation, and a real GDP reduction of 40% between 2000 and 2007 and a further 14% fall in 2008 [1]. An estimated 600,000 to 4.1 million Zimbabweans were food insecure [2]. Both commercial and subsistence farming were affected by the combination of land redistribution policies, unstable local currency, and government price controls.

Following the elections in March 2008, public services, including health care, suffered substantially. Most government health care facilities were closed between September 2008 and February 2009, during which time only a small number of mission hospitals, municipal primary care clinics, and private or non-governmental health care facilities continued to function [3]. This dual health service and economic collapse was accompanied by a widely publicized cholera epidemic [4], [5]; however, the status of other diseases remains unknown. Since February 2009 with the acquisition of the multicurrency system, the economy as well as food security have gradually improved [6].

It has been previously documented that a rise in HIV prevalence can lead to a rise in TB incidence as seen in Africa in the 1980s and 1990s [7]. Furthermore, household food insecurity has been identified as a risk factor for increased susceptibility to TB [8]. As Zimbabwe suffered from high HIV prevalence and widespread food insecurity during the crisis of 2008 and 2009, we undertook an ecological analysis to investigate the incidence of TB during the crisis years. Due to widespread closure of public health facilities during the peak of the crisis, there have been no previous reports on the incidence of TB in Zimbabwe during this time. We hypothesized that in the setting of high HIV prevalence, widespread food insecurity would lead to a rise in TB incidence in Zimbabwe.

## Methods

To investigate trends in tuberculosis (TB) incidence and HIV prevalence, we reviewed prospectively recorded databases from a northern Zimbabwean hospital [The Salvation Army Howard Hospital (HH)] between the years of 1995 to 2012. To confirm TB trends during the crisis, prospectively recorded cases of TB from an eastern Zimbabwean Hospital [Murambinda Mission Hospital (MH)] were also reviewed between the years of 2005 to 2009. Unlike the government hospitals, these mission hospitals receive external charitable support, which enabled them to function throughout the crisis. This provided a unique opportunity to study the effect of the crisis on tuberculosis incidence.

HH and MH are rural mission hospitals, located in the Mazowe district of northern Zimbabwe, and the Buhera district of eastern Zimbabwe, respectively. Both catchment populations are primarily agrarian.

Since 1995 HH prospectively maintained monthly disease-specific registries for TB, HIV/AIDS, and outpatient clinic diagnoses with basic demographic, and outcome data. Data for 2002 was incomplete due to damaged registries. MH maintained an electronic database of all TB cases between January 1, 2005 and May 31, 2009.

### *Tuberculosis*

Both hospitals followed national guidelines, which did not change throughout the entire study period. The diagnosis of TB was based on chronic cough, OR suggestive symptoms (fever, night sweats, weight loss), AND positive sputum smears and/or chest X-Ray findings with no response to broad spectrum antibiotics. There was no change in the diagnostic or laboratory techniques or the funding for diagnostic investigations or treatment at these institutions during the study years. There were no significant attempts in these districts to promote education to the communities regarding TB care and prevention during the study period. Staffing at the hospitals and food availability within the hospital did not change during the study period. Therapy was initiated by physicians at no cost using standard short course regimens. Directly observed therapy was provided for approximately 50% of MH patients with treatment otherwise self-administered. Sputum TB cultures and sensitivity testing were not routinely performed at the national laboratory in Bulawayo during the crisis period [9]. A single sputum specimen from a patient with prolonged treatment failure at HH in December 2007 was sent to a research laboratory in South Africa and found to contain Multi-Drug Resistant (MDR) TB.

### *HIV/AIDS*

HH Antenatal HIV results were prospectively collected from the HH Maternal Child Health (MCH) clinic from March 2001–April 2012 and was used as an indicator for the HIV seroprevalence in the population.

Baseline hemoglobin (Hgb) concentrations (2001–2008), and baseline CD4 counts, were obtained from all patients receiving antiretroviral (ARV) treatment at HH from 2001 to May 2012.

At HH outpatient clinic nutritional data on the number of cases of diagnosed nutritional deficiencies (Pellagra, Kwashiorkor, and Marasmus) and the number of cases of diarrhea both with and without dehydration were reviewed. The annual and monthly total number of outpatient visits to HH was reviewed to estimate changes in the community size.

Anonymized data was entered into Microsoft Excel spreadsheets, and analyzed using SPSS v20.0 and SAS version 9.2.

Incidence rates were calculated using the population of the local districts from the 2002, Zimbabwean census [10]. Gross domestic product (GDP) per capita, was obtained from the World Bank [11]. Measures of food insecurity were obtained from the Zimbabwe Food Security Outlook from the Famine Early Warning System of USAID [12].

Data was analyzed in an iterative manner to determine patterns and relationships between the variables of interest. An analysis of variance (ANOVA) with contrasts was applied to examine TB and HIV trends during the study period.

In the absence of local hospital ethics committees, permission for data review was obtained from hospital administrators and community leaders. This study was also submitted to the Research Ethics Board of the Senior Author in Canada (Lakeridge Health Corporation in Canada). The board considered the study and concluded that it constituted secondary use of anonymous information as described in section 2.4 of the Tri-council policy statement (TCPS2) on Ethical conduct for Research involving humans 2010 and therefore was exempt from REB review.

## Results

At HH a total of 11,560 cases of TB were treated between January 1^st^, 1995 and April 30^th^, 2012 (*Table 1*). Demographic data for TB patients was recorded at both locations. At HH, demographic information was taken from the first 200 TB cases from 2000–2011, excluding 2008, due to missing information. The male∶female ratio was 1.060∶1 and the mean age was 29.73 years (standard deviation (SD) = 17.52 years) and a range of 1 month to 93 years. 93.3% of cases were diagnosed as pulmonary tuberculosis and 6.7% extra-pulmonary tuberculosis.

**Table 1:** TB cases and TB cases/100,000 HH (1995–2012), MH (2005–2009), estimated national TB incidence reported to the WHO (1997–2011).

Year	HH TB cases	MH TB cases	HH TB cases per 100000	MH TB cases per 100000	Estimated national TB Incidence per 100000	National Case notifications per 100000	Estimated National Case Detection rate (%) (Low/High Estimates)	HH HIV seroprevalence In Antenatal Clinic	National HIV Prevalence
**1995**	352	+	176	+	+	264	55 (40/79)	+	
**1996**	443	+	222	+	+	+	57 (43/79)	+	
**1997**	503	+	252	+	540	+	63 (48/84)	+	
**1998**	794	+	398	+	562	+	61 (48/80)	+	27.30
**1999**	555	+	278	+	564	+	59 (47/76)	+	27.00
**2000**	477	+	239	+	586	407	56 (45/71)	25.92	26.20
**2001**	547	+	274	+	630	+	59 (48/74)	22.48	25.00
**2002**	+	+	+	+	683	+	59 (49/74)	22.19	23.60
**2003**	830	+	416	+	659	+	52 (43/66)	22.44	22.20
**2004**	899	+	451	+	674	+	55 (45/70)	19.34	20.70
**2005**	826	745	414	323	601	401	50 (41/63)	16.91	19.30
**2006**	680	888	341	385	557	+	45 (37/57)	17.45	18.10
**2007**	880	1013	441	439	782	+	43 (34/55)	16.34	17.10
**2008**	1108	1312	556	568	761	294	41 (33/53)	15.35	16.30
**2009**	1053	1266	528	548	740	344	52 (41/67)	11.17	15.70
**2010**	703	+	353	+	633	352	56 (44/72)	10.45*	15.20
**2011**	633	+	317	+	603	+	50 (40/65)	10.45*	14.90

Sources: national case notifications/100,000 [9] estimated national case detection rate [9], Howard Hospital HIV sero-prevalence in the Antenatal Clinic from 2000–2011 and National HIV Prevalence [11]. All incomplete data is given the symbol +.

* Data missing from end of 2010 and beginning of 2011. Data during these two years were therefore combined and averaged.

Overall, TB incidence rose significantly from 1995–2008, from 177/100,000 to 556/100,000 (*p*<.01). Tuberculosis incidence rose significantly from 177/100,000 in 1995 to 274/100,000 in 2001 (*p*<.05). Data in 2002 were incomplete, but there was a rise between 2001 and 2003 from 274/100,000 to 416/100,000 (*p*<.05). The rates subsequently stabilized until 2008 when there was a further rise of 34.6% to 556/100,000 as compared to rates from 2003–7 (*p*<.01). In 2009, TB rates remained high and not significantly changed with an incidence of 528/100,000.

At MH a total of 5,224 cases of TB were treated between January 1^st^, 2005 and December 31^st^ 2009 (*Table 1*). At MH the male∶female ratio was 1.036∶1 and the mean age was 34.36 years old (SD = 16.0), age range <1–100 years. The tuberculosis cases during this time consisted of 80% pulmonary tuberculosis and 20% extra pulmonary tuberculosis. Data from MH concurred that TB incidence rose between 2005–2007 (323/100,000 to 439/100,000) (p<0.01). There was a further 29.5% rise in 2008 to 568/100,000 (p<0.01) and rates remained elevated in the first 5 months of 2009 (577/100,000 on an annualized basis).

To assess whether the transfer of patients from closed government facilities in second half of 2008 to HH could account for the observed rise in TB incidence, we narrowed the data to look at the first 6 months of each of the years 2003–2007 (TB cases/month *mean* = 62.33, *SD* = 21.6) and compared this to the first 6 months of 2008 (before government facilities closed) (*mean* = 98.17, *SD* = 17.5). This analysis demonstrated a significant rise in TB incidence in early 2008 T(24) = 3.8 (*p*<.01). Similarly MH maintained detailed residence data on TB patients. For all analyses, only residents of the Buhera district were included, and therefore the rise in TB incidence was not related to patients coming from other districts.

After the crisis, the TB incidence at HH in 2010 and 2011, was 335/100,000 which was a 38% decline when compared to the crisis years of 2008 and 2009, when TB incidence was 542/100,000 (p<0.01). The most significant decrease seen was from 2009 to 2010, which saw a fall from 528/100,000 to 353/100,000 (p<0.001).

**Figure 1:**
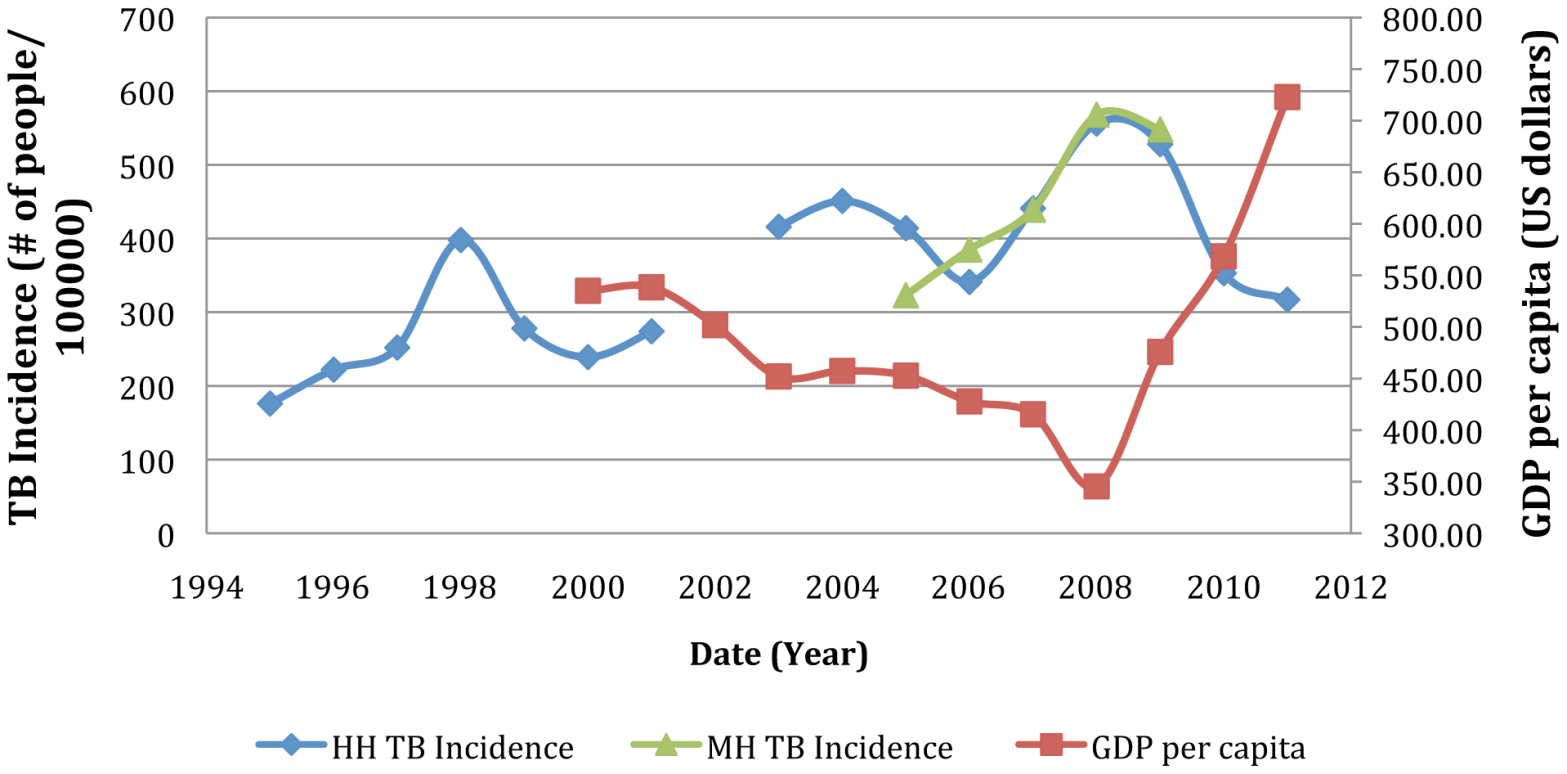
TB incidence at HH and MH versus GDP per capita in US dollars. GDP per capita is used to measure the average economic wellbeing of the Zimbabwean population. Source GDP per capita (In US dollars): World Bank [11].

**Figure 2:**
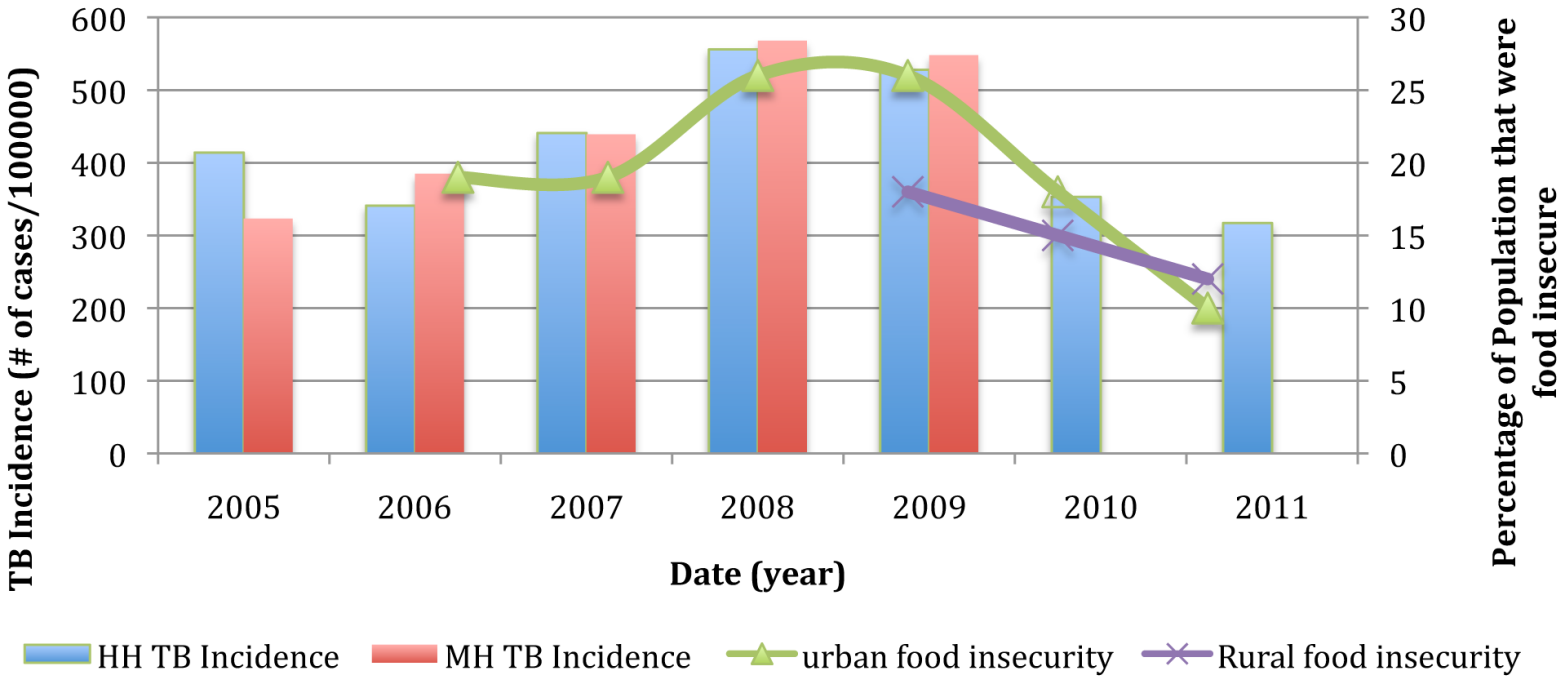
TB incidence at HH and MH versus rural and urban food insecurity. Consistent with the belief that food insecurity contributed to rise in TB, the figure shows an increase in food insecurity during the crisis and then a fall afterwards in urban environments. This parallels the rise in TB incidence during the crisis and the decrease seen after. Rural food insecurity was also seen falling after the crisis. Data for rural food insecurity during the crisis was not available. Source Urban and Rural food insecurity: Famine Early Warning Network [12].

TB incidence at HH and MH and the Gross Domestic Product (GDP) per capita as well as national food security data are displayed in figures 1 and 2 [11], [12].

TB incidence at HH (1995–2011) and MH (2005–2009) was tabulated by month of diagnosis. In both regions, TB incidence was significantly higher in months of food scarcity than in months of food abundance. In September–November (at the end of the dry season): (HH mean = 104.33 cases/month, SD = 18.32, MH mean = 65.71 cases/month, SD = 22.96) compared to immediately post harvest (April–June) (HH mean = 85.83 cases/month, SD = 25.64) (t (198) = 12.88, p<.001, MH mean = 56.12 cases/month, SD = 26.45) (t (96) = 23.36, p<0.001).

Outpatient data was analyzed annually and monthly to provide an indication of the population around HH. There was only a 3.5% rise in the number of outpatients between 2002 (59883) and 2008 (61978), with a rise after the crisis of 43% from 2008 to 2011 (108080). Seasonality was assessed using the monthly outpatient data. When comparing the dry season versus the post-harvest season the mean number of outpatient visits by season was not significantly different [19332 (SD = 3376) during the dry season and 19059 (SD = 2969) post harvest (t (7) = .260, p = 0.8)]. Although the total number of monthly or annual outpatient visits at MH was not documented, the percentage of TB cases, which were from the Buhera district versus outside the district, was documented. There was no change in this ratio with 20% of the cases being from outside the district both in 2005 and during the crisis years of 2008 and 2009.

Sputum TB cultures and sensitivity testing were not routinely performed at the national laboratory in Bulawayo during the crisis period [9]. A single sputum specimen from a patient with prolonged treatment failure at HH in December 2007 was sent to a research laboratory in South Africa and found to contain Multi-Drug Resistant (MDR) TB.

Antenatal HIV data from HH was available on 23,598 pregnant women tested from March 2001–April 2012, 3900 results were positive. The antenatal HIV registry between June 2010–April 2011 suffered environmental damage and could not be assessed. The number of pregnant women enrolled and the percent tested monthly (mean = 95.2%) were stable throughout. Antenatal HIV seroprevalence declined from March 1, 2001—May 31^st^ 2009 from 23% to 11% (F = 7.571, *p*<.001) (table 1). HIV seroprevalence continued to decline after the crisis as seen comparing March 2008–February 2009 (M = 15%, SD = 4%) with March 2011 to Feb 2012 (M = 8%, SD = 2%) (t(51) 2.69, p<.001).

Howard Hospital HIV Clinic CD4 counts at treatment initiation were available for 3,253 patients between January 2004 and May 2012. CD4 counts at ARV (Antiretroviral Treatment) initiation did not show any significant trends between the years before the crisis {2004 mean 150.9, *SD* = 200.0}, during the crisis {2009 mean 147.2, SD 94.0} and after the crisis {2011 mean 187.0, *SD* = 102.2}, (*p*>0.05). However there was a significant fall in mean hemoglobin at ARV initiation over time from 2004 (mean 11.01, *SD* = 1.92) to 2008 (10.6, *SD* = 1.7) (*p*<0.01). Data on hemoglobin at treatment initiation was not recorded at HH after 2008. Prior to 2008, data on HIV testing was not routinely linked with HH TB case logs. TB patients were routinely given pretest counseling and recommended to have HIV testing. From 2008 to 2011, the HIV seroprevalence has stayed relatively constant at 78%. Similarly at MH in 2008 75% of TB cases were HIV positive (see Table 2).

**Table 2:** HIV seroprevalence in TB patients at Howard Hospital (HH) (2008–2011) and Murabinda Hospital (MH) (2008) and nationally.

HIV+TB	TB Positive/Total tested at HH	Seroprevalence at HH	TB Positive/Total tested at MH	Seroprevalence at MH	Estimated National HIV Seroprevalence In TB Patients
**2008**	270/330	82%	1031/1394	75%	68%
**2009**	486/647	75%	+	+	52%
**2010**	317/428	74%	+	+	75%
**2011**	343/431	80%	+	+	60%
**Average**	**+**	**78%**	**+**	**75%**	**64%**

[9] Estimated National HIV seroprevalence in TB Patients. + Data unavailable.

At HH, all three measured nutritional deficiencies significantly increased over time (*p*<0.01 for each; *Table 3*). The prevalence of kwashiorkor most significantly increased between 2001 (130 cases) and 2008 (239 cases) (*p*<.01). The prevalence of pellagra also rose between 2001 (31 cases) and 2004 (118 cases) (p<0.01) and remained at this elevated level thereafter. Diarrhea, both with and without dehydration, increased over time (*p*<.01) (*Table 3*). There was however significant scatter within the data, and therefore this data should be interpreted with caution. Unfortunately no nutritional data after the crisis could be collected.

**Table 3:** Annual number of patients presenting with nutritional deficiencies or diarrhea at Howard Hospital.

Year	Pellagra	Kwashiorkor	Marasmus	Diarrhea (no dehydration)	Diarrhea (dehydration)
					
1995	69	122	+	803	89
1996	32	105	+	875	63
1997	22	85	+	778	44
1998	10	103	+	808	147
1999	11	180	+	816	199
2000	13	279	+	852	242
2001	31	130	+	927	325
2002	+	+	+	+	+
2003	14	574	+	1019	830
2004	118	321	+	1453	1251
2005	140	1168	431	2142	1406
2006	69	+	509	2482	1069
2007	117	333	369	2328	1019
2008	111	239	521	2789	1078
2009*	98*	523*	504*	2635*	1121*

*Note*, + denotes missing data.

* Data from January 1–May 31, 2009 estimate at annualized rate (×12/5).

## Discussion

This is the first study to suggest a rising TB incidence in the context of Zimbabwe's recent economic collapse. Our data also suggests that TB incidence has recently fallen back to pre-crisis levels now that the economy and food security have improved. To our knowledge, it is also the first to suggest an association between rising TB incidence and national economic decline in the absence of armed conflict. Even during the American Great Depression, TB incidence and mortality continued to decline [13], [14]. This distinction may be due to the high HIV seroprevalence in Zimbabwe, as the vast majority of TB cases in this region were HIV co-infected. This subset of the population may be particularly predisposed to immunologic compromise and increased susceptibility to TB in the setting of the food insecurity induced by economic decline

Several interactions between socioeconomic factors and TB prevalence in different regions of the world have been described [15], [16]. In sub-Saharan Africa, poverty has been associated with increased crowding and thus higher TB prevalence [17], [18]. TB has been associated with malnutrition in animal models as well as in humans [19]. In another high HIV prevalence African country (Zambia) lower socioeconomic position (SEP) was associated with higher TB prevalence and this association appeared to be mediated by malnutrition [8]. Our data suggests that during the recent Zimbabwean socioeconomic crisis, a fall in socioeconomic status, and thus rising malnutrition may have led to increased TB incidence on a national scale.

Our data from 1995–2007 correlated with the Zimbabwe trends of rising TB incidence reported to the World Health Organization (Table 1). Both the HH data and the estimated national incidence show a fall in TB incidence after the crisis in 2010. Our data demonstrated stable (HH) or rising (MH) rates between 2005–2007, and a further 35% increase at HH and a 29% increase at MH in 2008, remaining elevated in both centers in 2009. This rise in TB incidence seen both at HH and MH during the peak of the crisis in 2008–2009 was not seen in the estimated national incidence (Table 1). A rise in TB incidence in 2007, just prior to the peak of the crisis was estimated to have occurred in the nationally reported data, but changes during the crisis could not be adequately assessed due to a lack of functioning facilities during this time [9], [20]. This is suggested in table 1 where the National case detection rate during the crisis was estimated to have fallen from 50% in 2005 to 41% in 2008. Our data from HH and MH, which remained open throughout this time, suggest that TB incidence rose even further during the peak of the crisis. Following the crisis, the national case detection rate has been estimated to increase back to 56% in 2010. The data obtained from the two geographically distant mission hospitals, which continued to function throughout the crisis allowed for a consistent identification rate before, during and after the crisis. Therefore these results are likely a more reliable indicator of TB incidence. This finding provides an important insight into the health status of the population during this critical period.

Uncertainty regarding population denominators is a potential source of inaccuracy, applying to both local and national data. The population of the Mazowe district surrounding HH was 198,319 in the 1992 census and 194,927 by 2002 [10]. Subsequent to 2002, the economic crisis led to a dramatic increase in emigration from Zimbabwe to surrounding countries with estimates of 1.1 to 3 million people having left the country [21]. Nevertheless we used the more conservative population estimates from the population census of 2002 for the TB incidence calculations. Thus, both our local and the national TB estimates may understate the rise in incidence during the crisis. In regards to the population in the rural centers, there was a decrease seen during the crisis, from 2007 to 2008, and then an increase from 2008 to 2010 [22] (Table 4). Thus it is unlikely that the increase in TB during the crisis and the decrease of TB after the crisis is due to fluctuations in either the national or rural population. Further, it is unlikely that the increase in TB incidence is due to an increase in use of these hospitals due to the fact that outpatient visits at HH rose only 3.5% from 2002 to 2008 and thus is unlikely to account for the 35% increase in TB incidence seen at that time. Outpatient visit numbers rose from 2008 to 2011 by 46%. Therefore the post crisis fall in incidence was not likely to be due to patients leaving the catchment area. While MH did not keep track of outpatient data, the percentage of TB patients that came to the hospital from outside the Buhera district stayed consistent at 20% in 2005 and during the crisis years of 2008 and 2009. Since there was not a greater percentage of individuals outside the area that were diagnosed during the crisis we suspect that fluctuations of population were not likely to be leading to the measured changes in TB incidence.

**Table 4:** Zimbabwe Population, Rural Population, Urban percentage of population, and GDP per capita.

Year	Zimbabwe Population	Rural Population	Urban % of population	GDP per capita (In US dollars)
**1998**	12,229,500	8,200,173.26	33	523.49
**1999**	12,384,727	8,254,073.77	33	553.75
**2000**	12,503,652	8,282,669.16	34	535.04
**2001**	12,586,763	8,284,808.79	34	538.45
**2002**	12,640,922	8,267,314.68	35	501.71
**2003**	12,673,103	8,235,083.71	35	451.95
**2004**	12,693,047	8,194,681.92	35	457.38
**2005**	12,710,589	8,152,571.78	36	452.79
**2006**	12,724,308	8,103,602.79	36	427.83
**2007**	12,740,160	8,055,857.97	37	415.38
**2008**	12,784,041	8,025,565.26	37	345.41
**2009**	12,888,918	8,032,889.25	38	475.85
**2010**	13,076,978	8,090,726.29	38	568.43
**2011**	13,358,738	8,199,513.23	39	722.84

Data sources: GDP per capita (In US dollars) [11], Zimbabwe Population [20], Rural Population (Rural % of population) [20].

The rising incidence of TB between 2001 and 2003 parallels the onset of hyperinflation (inflation rate>100%/year) and a fall in per capita GDP in Zimbabwe (figure 1). We believe that food insecurity due to a lack of purchasing power may have contributed to the rise in TB rates between 2001 and 2003. The collapse of the economy in 2008 may have led to even further restrictions in nutrition. A survey in 2008/2009 found an increase in urban food insecure households to 26% as compared to 19% in 2006/2007 [12] (figure 2). Since the crisis average monthly per capita GDP has steadily increased from $44 per month in 2009 to $58 per month in 2011, which has increased the purchasing power for food consumption [12] (figure 1). Food insecurity has decreased substantially from the peak of the crisis, from 26% in urban centers in 2008/2009 to 10% in urban centers in 2011/2012 (figure 2). Similarly there was a decline in food insecurity in rural centers from 18% in 2009/2010 to 15% in 2010/2011, with a predicted food insecurity prevalence of 12% in 2011/2012 [12]. Thus the decreasing incidence of TB post-crisis occurred at the same time as there was a decline in the proportion of food insecure individuals in Zimbabwe (figure 2).

A seasonal trend in TB incidence with a rise in late winter/early spring has been described in both the northern and southern hemispheres [23], [24]. In the United Kingdom and in Australia [25], [26], this rise was seen in summer and has been attributed to a delayed effect of vitamin D deficiency from dark winters leading to a rise in TB rates in summer [25], [26]. On the other hand, tuberculosis data from the United States has suggested that the seasonality of tuberculosis may not be due to effects of vitamin D levels due to a lack of variability of TB rates amongst the latitudes in the United States [27]. Instead the pattern is attributed to an increase of transmissions during the winter, which leads to a greater frequency of diagnosis of acute cases in the spring [27]. In South Africa's Western Cape, this seasonality is thought to be related to cool, wet winters leading to crowding in poorly ventilated homes [24]. The climate of Northern and Eastern Zimbabwe, however, is quite different in that the rains occur in the summer, between late November and early March [28]. The southern winter in Zimbabwe is the dry season, and as temperatures remain mild [28], time spent indoors may not increase during this period. Furthermore, due to cloud cover in summer there are more hours of sunlight in the winter [28]. Therefore, we suspect that the seasonal effect seen in our study is more likely related to nutritional factors associated with harvest time, unlike the trends seen in countries with more variable temperature conditions. As most patients in the rural areas are subsistence farmers, their food supply is dependent on the harvest. Without artificial irrigation, a single crop becomes available in March. Nutrition is best at this time, with food stocks at a minimum at the start of the next rainy season. We suspect that the immune compromise associated with malnutrition leads to a seasonal incidence of reactivated TB. The dramatic rise in TB incidence seen during the Zimbabwean economic crisis, with its associated rise in food insecurity, coupled with long term seasonal trends related to the harvest, lead us to suspect that TB incidence in Zimbabwe is linked to nutritional factors. Further, the fact that there was no significant difference in the number of outpatient visits between the dry and post-harvest season supports our conclusion that food insecurity was responsible for the increase in TB incidence rather than a seasonal trend in the community availability to seek medical attention.

We do not believe that an increased prevalence of HIV led to the recent rise in TB incidence in 2008, although it is likely involved in the rise experienced between 1995 and 2001. Our local antenatal prevalence data demonstrate an ongoing decline in HIV prevalence in the HH community from March 2001—May 2009, which unlike the TB data, matches national trends. This decline from peak HIV incidence in 1997 [29]–[31] has been documented in previous Zimbabwean studies and has been attributed to public health interventions, safer sex, and the addition of ARV drugs [30], [31].

The relationship between socioeconomic status and HIV prevalence in women in sub-Saharan Africa is quite complex and has been reviewed in detail [32]. In some low-income countries, where poverty is widespread, increasing access to resources for women may initially increase the risk of HIV or have no effect on risk-taking behaviors. On the other hand in other countries where per capita income is higher and inequalities in wealth are greater, increasing socioeconomic status may decrease HIV risk [32]. At HH HIV prevalence in pregnant women appears to have continued to fall despite great changes in socioeconomic status during and after the crisis years.

It is possible that, with the maturation of the HIV epidemic, there are a greater proportion of HIV-infected persons with advanced immunosuppression, thus contributing to the increase in TB cases seen in 2008/2009. This is compatible with HIV prevalence rates remaining high in TB patients, unlike antenatal clinic attendees. Although there has been a rollout of antiretroviral therapy over this time in our community, high coverage rates are required to decrease the incidence of TB [33]. The local rollout of ARVs was inhibited by transport, human resources and other logistical problems both prior to and during the 2008/2009 crisis and thus, was likely inadequate to impact TB incidence during that time. However, peak HIV prevalence in Zimbabwe occurred in 1997 [29]–[30] making the 2008/2009 rise in TB incidence unlikely to be related to changes in the HIV epidemic [33]. The lack of significant changes in CD4 counts of patients at ARV enrollment from 2004 to 2011 suggests that the stage of immunosuppression at which patients present has not changed substantially. The fall in average hemoglobin levels seen from 2004–2008 in HIV patients at ARV enrollment over time suggests that nutritional deficiencies associated with economic decline may have been etiologically important.

A limitation of the data from HH and MH is that the diagnosis of TB was primarily based on clinical, smear positivity and radiological data, rather than culture confirmation. However the diagnostic algorithm used at HH and MH is compatible with WHO guidelines for TB diagnosis in resource limited countries with high HIV prevalence [34]. The fact that the same trends of TB incidence leading up to and during the crisis are seen at both study sites, which are located in different areas of the country, strengthens our conclusions.

The lack of culture confirmation also applies to the national data due to the non-functioning of the national reference laboratory during this time [20]. Our mean smear positivity rate using pooled HH and MH data was 34.4%, which was similar to the Zimbabwean national rate of 32% [9]. This suggests that similar diagnostic approaches were being used in our study sites and other Zimbabwean institutions. Though clinical criteria may lead to under- or over- diagnosis of TB, there was no difference in clinical algorithms or the clinical threshold for initiating diagnostic workups for tuberculosis over the period of the study. Therefore, we believe it is plausible that the prospectively recorded trends in TB cases at these mission hospitals can be used to reflect underlying trends in Zimbabwean national TB incidence during the peak crisis years.

Another limitation is the ecological nature of this study. A population-based study would be a useful addition, as it would take into account local movement of people and patients. This would be necessary to confirm these findings, however it is difficult to predict these events happening in advance and thus an ecological study would be more practical. In addition, this study is limited due to the fact that hospital based data from two locations were used as indicators of national trends and therefore are subject to unknown confounding variables. These hospitals were two of the few health centers open during the crisis so they may be the best indicators available.

The use of pregnant women as the measure of HIV seroprevalence may also be a limitation of the study as this population may not be representative of the HIV prevalence in the rest of the population, However, since the same population was used for the entire study and the trends are similar to national data, this suggests that the sample may be fairly representative.

As well, there was frequent movement throughout Africa during and after the crisis and very few resources to accurately keep track of this movement. There also was to and fro movement between the rural and urban areas during the crisis. In order to minimize the impact of this limitation, we were fairly conservative with our population denominators when calculating TB incidence. Outpatient visit attendance data obtained from HH and MH suggests that the changes in TB incidence were unlikely to be due to changes in the total number of patients accessing the facilities during the crisis.

This study relates the impact of economic collapse to TB case numbers at two mission hospitals. Although true incidence rates are difficult to estimate when the number of residents in the area are elastic, in the absence of more robust nationally or regionally derived data this may be the best available information for the estimation of the regional or national TB rates. However, as discussed above, these data are subject to many confounders, and their significance should be interpreted with some caution. Nonetheless, this study suggests that in Zimbabwe both seasonal and overall food insecurity are associated with rising TB incidence. The implications of this study are that economic collapse with resultant food insecurity and malnutrition in the setting of high HIV seroprevalence may dramatically raise TB incidence. The return of TB incidence to baseline levels associated with economic recovery is reassuring. The nutritional and economic predictors of TB incidence require further study and our findings would be strengthened by population-based and nationally derived data.
